# Human papillomavirus type 18 DNA and E6-E7 mRNA are detected in squamous cell carcinoma and adenocarcinoma of the lung.

**DOI:** 10.1038/bjc.1995.69

**Published:** 1995-02

**Authors:** I. Kinoshita, H. Dosaka-Akita, M. Shindoh, M. Fujino, K. Akie, M. Kato, K. Fujinaga, Y. Kawakami

**Affiliations:** First Department of Medicine, Hokkaido University School of Medicine, Japan.

## Abstract

**Images:**


© 1995 Stockton Press All rights reserved 0007-0920/95 $9.00

### Human papillomavirus type 18 DNA and E6-E7 mRNA are detected in squamous cell carcinoma and adenocarcinoma of the lung

I Kinoshita[1], H Dosaka-Akita[1], M Shindoh [2,3], M Fujino[1], K Akie[1], M Kato[1], K Fujinaga[3] and Y Kawakami[1]

[1]*First Department of Medicine, Hokkaido University School of Medicine;* [2]*Department of Oral Pathology, Hokkaido University School of Dentistry;* [3]*Department of Molecular Biology, Cancer Research Institute, Sapporo Medical University, Sapporo 060, Japan.*

**Summary** To provide an accurate evaluation of the association of human papillomavirus (HPV) with lung cancer, 36 cases of lung cancer were analysed for HPV DNAs by polymerase chain reaction (PCR) with dot-blot and Southern blot analyses, and for the transcripts from the E6-E7 transforming region by *in situ* hybridisation (ISH). HPV-18 DNA was detected in three (8%) of 36 specimens; histologically, in one (10%) of 10 squamous cell carcinomas and two (9%) of 22 adenocarcinomas. Neither HPV-16 nor -33 DNA was detected in any cases examined. Expression of E6-E7 mRNA was confirmed in the cases which contained HPV-18 DNA. HPV-18 may play an important role in the development and progression of cancer in some cases of both squamous cell carcinoma and adenocarcinoma of the lung.

**Keywords**: HPV; lung cancer; PCR; ISH

Human papillomaviruses (HPVs) are a heterogeneous group of double-stranded, non-enveloped DNA viruses with over 60 genotypes (for review, see de Villiers, 1989). They cause benign epithelial proliferations, but some types of HPV have been implicated in the pathogenesis of carcinomas for many sites, including the anogenital area, oropharynx and upper respiratory tract (Shindoh *et al.*, 1992; Stoler *et al.*, 1992; Anwar *et al.*, 1993). In particular, more than 90% of cervical carcinomas contain HPV DNAs that usually correspond to HPV-16 and HPV-18, so-called 'high-risk HPVs', and, less frequently, HPV-31, -33 and -35 (for review, see zur Hauzen, 1991).

High-risk HPV DNAs are frequently integrated into cellular DNA in these cancers, and the long-control region and the E6 and E7 regions of the HPV genome are preferentially conserved, although other regions of the genome are often deleted (Schwarz *et al.*, 1985; Takebe *et al.*, 1987). The E6 and E7 regions of high-risk HPVs can immortalise primary human cervical, epidermal, mammary and even bronchial epithelial cells (Woodworth *et al.*, 1988; Hawley *et al.*, 1989; Band *et al.*, 1990; Willey *et al.*, 1991). The E6 and E7 proteins of high-risk HPVs bind to the proteins encoded by the host tumour-suppressor genes, the p53 and retinoblastoma susceptibility (RB) genes respectively, to disrupt their normal function for controlling the cell cycle (Munger *et al.*, 1989; Scheffner *et al.*, 1990; Werness *et al.*, 1990).

Recent studies revealed that the p53 gene is mutated in 73% of small-cell lung cancers (SCLCs) and 45% of non-small cell lung cancers (NSCLCs) (Chiba *et al.*, 1990; Takahashi *et al.*, 1991) and that the RB gene is inactivated in 60-100% of SCLC cell lines and 32% of primary NSCLCs (Harbour *et al.*, 1988; Yokota *et al.*, 1988; Reissmann *et al.*, 1993). Because alterations of the p53 and RB genes are involved in many lung cancers, pulmonary infection with high-risk HPV might play an important role in the development and progression of lung cancer through the inactivation of the p53 and RB genes.

In lung cancer, HPV DNAs have been recently identified by *in situ* DNA hybridisation and Southern blot hybridisation in several per cent of cases of squamous cell carcinomas but neither in small cell carcinoma nor in adenocarcinoma thus far (Stremlau *et al.*, 1985; Ostrow *et al.*, 1987; Syrjänen *et al.*, 1989; Bejui *et al.*, 1990; Yousem *et al.*, 1992). To provide a more accurate evaluation of the association of HPV with lung cancer, we examined the existence of HPV DNA in lung cancer tissues by a polymerase chain reaction (PCR) method and dot-blot hybridisation, and confirmed it by analysing the PCR product with Southern blot hybridisation and the expression of E6-E7 mRNA with *in situ* hybridisation (ISH). Furthermore, the mechanisms involved in pulmonary carcinogenesis associated with HPV are discussed.

Correspondence: H Dosaka-Akita, First Department of Medicine, Hokkaido University School of Medicine, North 15, West 7, Kita-ku, Sapporo 060, Japan
Received 6 April 1994; revised 9 September 1994; accepted 20 September 1994

### Materials and methods

### *Tissue specimens and DNA preparation*

Tissue from 36 lung cancers was obtained by surgical resection or autopsy from Hokkaido University Hospital, Sapporo Minami-Ichijo Hospital and National Sapporo Minami Hospital during 1988 and 1992. Samples were taken 1-3 h after death in autopsy cases. All samples were divided into two parts, one frozen in liquid nitrogen and stored at $-80°C$ for DNA preparation and the other routinely fixed in formalin and embedded in paraffin for haematoxylin and eosin staining and ISH. According to the 1981 WHO classification, they were diagnosed histopathologically as adenocarcinoma ($n = 22$), squamous cell carcinoma ($n = 10$), small-cell carcinoma ($n = 2$), adenosquamous carcinoma ($n = 1$) and metastatic lung cancer of tongue carcinoma ($n = 1$). Genomic DNA was isolated by proteinase K digestion followed by phenol chloroform extraction and ethanol precipitation (Maniatis *et al.*, 1989).

### *Primers and probes for polymerase chain reaction (PCR)*

Type-specific primers and probes were synthesised on a DNA synthesiser (MilliGen Bioresearch, Brulington, USA) on the basis of published sequences of HPV-16, -18 and -33, as previously described (Shimada *et al.*, 1990). In brief, the primer p16-1 (5'-AAGGGCGTAACCGAAATCGGT-3') was located close to the 5' end of the E6 sense sequence of HPV-16, which was available as a common primer of HPV-16, -18 and -33. The primers p16-2R (5'-GTTTGCAGCTCT-GTGCATA-3'), p18-2R (5'-GTGTTCAGTTCCGTGCACA-3') and p33-2R (5'-GTCTCCAATGCTTGGCACA-3'), corresponding to type-specific E6 antisense sequences of HPV-16, -18 and -33 respectively, were located in the middle of E6 open reading frame (ORF). The oligonucleotide probes pB16-I (5'-CATTTTATGCACCAAAAGAGAACT-GCAATG-3'), pB18-I (5'-TGAGAAACACACCACAATAC-TATGGCGCGC-3') and pB33-I (5'-CATTTTGCAGTAAG-GTACTGCACGACTATG-3') were also type-specific for HPV-16, -18 and -33 respectively, and located in the middle of amplified regions.

*PCR and dot-blot hybridisation*

PCR was performed essentially as described by Shimada *et al.* (1990). Cloned HPV-16, -18 and -33 plasmid DNA were used as positive control templates for PCR. Each cloned HPV DNA or cellular DNA (500 ng) was denatured at 94°C for 10 min, and placed in the reaction mixture (50 μl) containing 10 mM Tris-HCl, pH 8.3, 50 mM potassium chloride, 1.5 mM magnesium chloride, 200 μM each dNTP, 100 $\mu g\ ml^{-1}$ gelatin, 2.5 units of *Taq* polymerase and 0.2 μM of each primer. The mixture was subjected to 35 cycles of amplification using a DNA thermal cycler (Perkin-Elmer Cetus, Norwalk, CT, USA). Each cycle included denaturation at 94°C for 1 min, annealing at 55°C for 2 min and extension at 72°C for 2 min. To avoid false-positive results, a reagent control (no template DNA) was included with each amplification. A 1-μl aliquot of the reaction mixture was dotted onto a nylon membrane filter (Biodyne Nylon Membranes, Pall BioSupport, East Hills, USA) and hybridised with each $^{32}P$ end-labelled HPV type-specific oligonucleotide probe by dot-blot analysis. Filters were washed twice in 0.2 × SSC, 0.1% SDS, at 55°C for 20 min. Finally, the filters were autoradiographed using Kodak XAR-5 film (Kodak, Rochester, NY, USA).

*Southern blot hybridisation*

The amplified products of the three HPV-18-positive cases and four HPV-18-negative cases in PCR and dot-blot analyses were further confirmed by Southern blot analysis. A 1.5% agarose gel in which 20 μl of the PCR products was separated by electrophoresis was denatured in 0.2 N sodium hydroxide, 0.6 M sodium chloride and neutralised in 1 M Tris-HCl, pH 7.5, 0.6 M sodium chloride. The amplified products were transferred to a nylon membrane filter and cross-linked using ultraviolet exposure. The filter was incubated in prehybridisation solution consisting of 50% formamide, 5 × SSPE, 0.3% SDS, 10 × Denhardt's solution and 250 μg $ml^{-1}$ herring testis DNA at 42°C overnight. HPV-18 DNA probe was labelled with [α-$^{32}$P]dCTP (3000 Ci $mmol^{-1}$, Amersham, Tokyo, Japan) by random priming and added to prehybridisation solution. Hybridisation was carried out at 42°C for 16 h. The filter was washed twice in 0.2 × SSC, 0.1% SDS, at 60°C for 20 min and then autoradiographed using Kodak XAR-5 film.

In situ *hybridisation (ISH)*

ISH for the detection of the E6-E7 mRNA of HPV-18 using a riboprobe was performed in HPV-18-positive cases essentially as descsribed by Stoler *et al.* (1992). In brief, an HPV-18 clone in pBR322 was subcloned into the transcription vector pGEM3 (Promega, Madison, WI, USA) as a subgenomic clone corresponding to the E6-E7 open reading frames (ORFs) [*Bam*HI (120) to *Hinc*II (658)]. The plasmid was linearised with *Bam*HI (Takara, Osaka, Japan) or *Hinc*II (Takara) and then an antisense or sense probe was synthesised with SP6 or T7 RNA polymerase (Boehringer Mannheim, Mannheim, Germany) respectively, in the presence of digoxigenin-UTP (Boehringer Mannheim) or [$^{35}$S]UTP (> 1000 Ci $mmol^{-1}$, Amersham). Following the probe synthesis, the template DNA was digested with RNAse-free DNAse (Boehringer Mannheim), and the probe was subjected to limited alkaline hydrolysis and reduced to an average size of 200 nucleotides.

Five-micron-thick tissue sections mounted on APS-coated glass slides (Matsunami Glass, Japan) were deparaffinised in xylene and rehydrated through a graded series of ethanol. Sections were treated with 0.2 N hydrochloric acid at room temperature for 10 min, 10 $\mu g\ ml^{-1}$ proteinase K at 37°C for 10 min, refixed with 4% paraformaldehyde and then acetylated with 0.25% (v/v) acetic anhydride. Slides were dehydrated through a graded series of ethanol and air dried. Hybridisation was carried out at 37°C for 16 h in humid conditions ($T_m$ − 20°C). The probe concentrations were 0.8 $\mu g\ ml^{-1}$ for a digoxigenin-labelled probe and $1 \times 10^7$ c.p.m. $ml^{-1}$ for a $^{35}$S-labelled probe. After hybridisation, slides were washed under high-stringency conditions ($T_m$ − 10°C). For digoxigenin-labelled probe, immunodetection was carried out by using a DIG ELISA Detection Kit (Boehringer Mannheim). In brief, sections were incubated with alkaline phosphatase conjugated antidigoxigenin antibody diluted 1:500 in 0.1 M Tris-HCl, pH 7.5, 0.15 M sodium chloride for 30 min. After washes, slides were immersed in a mixture of nitroblue tetrazolium (NBT) and 5-bromo-4-chloro-3-indolyl phosphate (BCIP) solution overnight and counterstained with methyl green. For a $^{35}$S-labelled probe, slides were overlaid with Konika NRM-2 autoradiography emulsion (Konika, Tokyo, Japan), exposed for 3 weeks at 4°C, developed photographically and counterstained with haematoxylin and eosin.

For controls, a known HPV-18 E6-E7 mRNA-positive cell line (HeLa) and a known HPV-negative cell line (C-33A) (Schwarz *et al.*, 1985; Yee *et al.*, 1985) were grown on chamber slide glass (Nunc, Naperville, USA), fixed with 4% paraformaldehyde in phosphate-buffered saline (PBS) for 30 min, and then processed for ISH.

### Results

We performed the PCR using HPV type-specific primers and dot-blot hybridisation assay using HPV type-specific probes to elucidate the role of HPV in the development and progression of lung cancer. HPV-18 DNA was detected in three (8%) of 36 specimens; histologically, in one (10%) of 10 squamous cell carcinomas and two (9%) of 22 adenocarcinomas (Table I). The results of the PCR and dot-blot hybridisation of HPV-18 in 19 cases are shown in Figure 1a. Neither HPV-16 nor HPV-33 DNA was detected in any case examined (Figure 1b and data not shown).

The above result on HPV-18 by the PCR and dot-blot analyses was confirmed by Southern blot analysis following PCR. As shown in Figure 2, an expected 140 bp band was observed in the HPV-18-positive cases in the dot-blot analysis. No positive signals were observed in HPV-18-negative cases.

In the HPV-positive cases, we performed ISH using digoxigenin-labelled riboprobes corresponding to the E6-E7 region of HPV-18 to confirm that the result of PCR was not false positive and that the tumour cells in fact expressed mRNA of the E6-E7 transforming region.

An HPV-positive cell line (HeLa) and an HPV-negative cell line (C-33A) were used as controls. Strong cytoplasmic signals with the E6-E7 antisense probe and no signals with the sense probe were observed in HeLa cells (Figure 3a and data not shown). No hybridised signals with either probe were detected in C-33A cells (Figure 3b and data not shown).

The case of squamous cell carcinoma that was HPV-18-positive in PCR analysis (case 1 in Table II) exhibited abun-

**Table I** Detection of HPV DNA in lung cancers

| | *No. of cases* | *HPV DNA type* 16 | 18 | 33 |
|---|---|---|---|---|
| Squamous cell carcinoma | 10 | 0 | 1 | 0 |
| Adenocarcinoma | 22 | 0 | 2 | 0 |
| Adenosquamous carcinoma | 1 | 0 | 0 | 0 |
| Small cell carcinoma | 2 | 0 | 0 | 0 |
| Metastatic lung cancer of tongue carcinoma | 1 | 0 | 0 | 0 |
| Total | 36 | 0 | 3 | 0 |

**Figure 1** Detection of HPV DNA using the PCR and dot-blot hybridisation methods described in the Materials and methods section. In this set of dot blottings, 19 amplified DNA fragments from frozen tissue samples were dotted on to three different membranes, and each membrane was hybridised to a type-specific probe for HPV-16, -18 or -33. **a**, Hybridisation with probe pB18-I for HPV-18. Two samples are positive (case 1 and case 14). Controls are dotted in row 3. Columns 1, 2 and 3 are positive controls of cloned HPV-16, -18 and -33 plasmid DNAs respectively. NC (column 4) is a negative control (no template DNA). **b**, Hybridisation with probe pB16-I for HPV-16. No sample was positive. For controls, see **a**. No HPV-33 DNA was detected (data not shown).

**Figure 2** Southern blot analysis of the PCR products described in the Materials and methods section. After gel electrophoresis, the products were Southern blot transferred and hybridised with a $^{32}$P-labelled HPV-18 DNA probe. Lanes 1–3, the HPV-18-positive cases in dot-blot analysis; lanes 4–7, HPV-18-negative cases in dot-blot analysis; lanes 8, 9 and 10, cloned HPV-16, -18 and -33 plasmid DNAs, respectively, for controls. The amplified product was confirmed to show an expected 140 bp band in the HPV-18-positive cases. φX 174 DNA/*Hae*III digest was used as a size marker.

dant cytoplasmic hybridised signals and a trace of nuclear signals with the E6–E7 antisense probe in cancer cells except in some areas of keratinisation, whereas stromal cells showed no hybridised signals (Figure 4a). The abundant cytoplasmic signals were considered to represent a high level of the E6–E7 gene expression while the trace nuclear signals might represent untransported mRNA precursors or be from intron sequences removed from E6*–E7 mRNA. This finding was

**Figure 3** *In situ* hybridisation with a digoxigenin-labelled E6–E7 antisense probe in controls. **a**, HPV-18-positive HeLa cell. Strong cytoplasmic hybridised signals can be observed (bar = 50 μm). **b**, HPV-negative C-33A cell. No hybridised signals can be observed (bar = 100 μm).

confirmed by the control hybridisation experiment with the sense probe, which abolished the hybridised signals (Figure 4b). One case of adenocarcinoma with HPV-18 DNA in PCR analysis (case 2 in Table II) showed cytoplasmic hybridised signals with the antisense probe (Figure 5a). These signals were also abolished with the sense probe (Figure 5b).

A definite signal was not observed by this ISH method in the other case of adenocarcinoma (case 3 in Table II). However, since case 3 showed poor preservation of morphology, the RNA in this specimen was thought to have degenerated too much for the digoxigenin-labelled probe to detect the HPV E6–E7 message. In this case, cytoplasmic hybridised signals were weakly observed by ISH using a $^{35}$S-labelled antisense probe corresponding to the same E6–E7 region to detect lesser amounts of transcripts sensitively (data not shown). Finally, we believe that not only HPV-18 DNA but also expression of the E6–E7 viral oncogene was detected in case 3, even though the transcript level was decreased by degeneration.

Characteristics of the HPV-positive cases were analysed and are shown in Table II. Strikingly, all the patients were male smokers. Pathologically, case 1 was moderately differentiated squamous cell carcinoma without koilocytotic feature. Case 2 was moderately differentiated acinar adenocarcinoma in which cytoplasmic mucin and mitosis were often observed. Case 3 was well-differentiated papillary adenocarcinoma. In the HPV-positive cases, adjacent bronchi were examined for dysplasia, metaplasia, papillamatous lesion and koilocytotic feature. However, none of these findings could be observed in each case.

### Discussion

In the present study, we detected HPV-18 DNA in one (10%) of 10 squamous cell carcinomas and two (9%) of 22 adenocarcinomas by the PCR method. Detection of high-risk HPV in adenocarcinoma of the lung has not been reported thus far. Several investigators have identified HPV-16 and -18 DNA in 3–12% of squamous cell carcinomas and in one anaplastic carcinoma of the lung by *in situ* DNA hybridisation and Southern blot hybridisation (Stremlau *et al.*, 1985; Ostrow *et al.*, 1987; Syrjänen *et al.*, 1989; Bejui *et al.*, 1990; Yousem *et al.*, 1992). Our detection rate of high-risk HPV in squamous cell carcinomas was compatible with previous studies.

Two recent studies used PCR-based assays to screen for HPVs in lung cancers and found none (Shamanin *et al.*,

**Table II** Characteristics of HPV-18-positive lung cancers

| | *Case 1* | *Case 2* | *Case 3* |
|---|---|---|---|
| Age (years) | 58 | 72 | 72 |
| Sex | Male | Male | Male |
| Pack-years of smoking[a] | 27 | 45 | 45 |
| Pathological stage | IV (Operation) | IV (Autopsy) | I (Operation) |
| Histology | Squamous cell carcinoma | Acinar adenocarcinoma | Papillary adenocarcinoma |
| Degree of differentiation | Moderate | Moderate | Well |
| Tumour | | | |
| Koilocytosis | – | – | – |
| Dyskeratosis | + | – | – |
| Adjacent bronchi | | | |
| Dysplasia | – | – | – |
| Metaplasia | – | – | – |
| Koilocytosis | – | – | – |

[a]Pack-years of smoking were calculated as number of years of smoking × average number of packs smoked per day.

**Figure 4** *In situ* hybridisation with digoxigenin-labelled E6–E7 probes in a case of squamous cell carcinoma which was HPV-18 positive in the PCR analysis. **a**, Antisense probe. Abundant cytoplasmic hybridised signals and a trace of nuclear signals can be observed in cancer cells except in some areas of keratinisation. Stroma cells had no hybridised signals (bar = 100 μm). **b**, Sense probe. The signals hybridised with the antisense probe were abolished in the adjacent section (bar = 100 μm).

**Figure 5** *In situ* hybridisation with digoxigenin-labelled E6–E7 probes in a case of adenocarcinoma which was HPV-18 positive in PCR analysis. **a**, Antisense probe. Cytoplasmic hybridised signals can be observed in adenocarcinoma cells (bar = 100 μm). Anthracotic lung tissue (arrow) can also be observed in the lung cancer tissue. **b**, Sense probe. The signals hybridised with the antisense probe were abolished in the adjacent section (bar = 100 μm).

1994; Szabó et al., 1994). However, both of the published papers used so-called consensus primers of PCR to cover a broad spectrum of HPVs, and the sensitivities of their assays were one genome copy per cell and one genome copy per ten cells respectively. We used type-specific primers and probes for detecting high-risk HPVs, and this method can detect one copy per $10^5$ cells (Shimada et al., 1990). Furthermore, Shamanin et al. (1994) used the primers in the L1 region, which is often deleted for integration in cancer cells, while we used the primers in the E6 region, which is always conserved for the integration (Takabe et al., 1987). Therefore, we could minimise false-negative results compared with these two studies.

Although ISH is less sensitive than PCR, it makes it possible to examine viral expression in infected cells and the relationship between pathological changes and the presence of HPV. With this method, we have demonstrated expression of E6–E7 mRNA in lung cancer. This ISH result revealed not only the presence of the HPV genome but also the expression of the viral oncogene, suggesting that they play a causative role in the development and progression in these tumours. Furthermore, the absence of E6–E7 mRNA in some areas of keratinisation in squamous cell carcinoma suggested the presence of cellular control mechanisms for viral mRNA transcription that might be related to the state of differentiation of the cell as previously reported (Higgins et al., 1991).

It is noteworthy that we detected HPV-18 in two cases of adenocarcinoma, because HPV has been thought to infect squamous cell metaplasia but not native columnar epithelium, and thus not to be involved in the genesis of adenocarcinoma of the lung (Bejui et al., 1990). However, in cervical cancer, high-risk HPV is known to be involved in the carcinogenesis of three major histological cell types: squamous, glandular and small-cell types (Stoler et al., 1992). Whereas HPV-16 is the most prevalent virus infecting the uterine cervix and is closely associated with squamous cell carcinomas, and sometimes with adenocarcinomas, HPV-18 is most consistently associated with adenocarcinomas and small-cell neuroendocrine carcinomas of the cervix and less frequently with squamous cell carcinomas (Tase et al., 1988; Stoler et al., 1991). Together with these findings, our findings suggest that HPV-18 can be involved in the pathogenesis of adenocarcinoma of the lung as well as that of the cervix.

Stoler et al. (1992) found that most HPV-16 DNAs existed in a mixed episomal and integrated state, whereas all HPV-18 DNAs existed in an integrated state, regardless of the cell type. Therefore, they suggested three possibilities with regard to the virus–host relationship, none of which were mutually exclusive: first, the virus infected a multipotent precursor cell; second, the virus preferentially infected a cell already committed to a certain cell type; and, third, the virus exerted some influence on cell differentiation. Their suggestions and our present findings that all three HPV-positive patients were smokers indicated that HPV might infect multipotent basal cells in the bronchial epithelium at sites of microabrasion caused by smoking, or at the border of the metaplastic mucosa, equivalent to the transitional zone of the uterine cervix. Through these possible mechanisms, high-risk HPVs, especially HPV-18, are thought to be involved in the development and progression of both squamous cell carcinoma and adenocarcinoma of the lung.

We only detected HPV-18, although previous studies reported that HPV-16 was more prevalent than HPV-18 in lung cancer in Western countries (Stremlau et al., 1985; Ostrow et al., 1987; Syrjänen et al., 1989). This difference might be caused by geographic or racial factors, as was shown in laryngeal cancer (Anwar et al., 1993).

In a recent study it was shown that bronchial papillomas associated with HPV-16 and or -18 are at high risk for the development of squamous cell carcinoma (Popper et al., 1992). However, we could not find a papillomatous lesion in the neighbouring area of the HPV-positive squamous cell carcinoma. We suggest that not all squamous cell carcinomas associated with HPV-18 arise from the benign papillomatous lesion, which is consistent with the observation reported by Syrjänen et al., (1989) that only four of 12 HPV DNA-positive bronchial squamous cell carcinomas showed lesions, including papilloma, suggestive of HPV infection.

p53 and RB proteins have been reported to be inactivated by the E6 and E7 proteins of high-risk HPVs *in vitro* (Munger et al., 1989; Scheffner et al., 1990; Werness et al., 1990). It also has been reported that high-risk HPVs cooperate with an activated ras oncoprotein to transform baby rat kidney cells (Storey et al., 1988), and that E7 protein of high-risk HPV overcomes the inhibition of c-*myc* expression by transforming growth factor $\beta_1$ (Pietenpol et al., 1990). We examined the three HPV-positive lung cancers for the mutation of p53 by the PCR-based single-strand conformation polymorphism (SSCP) analysis of exon 5 to exon 9 and found p53 mutation in all three cases (in preparation). This finding suggests that the cellular target for HPV is not p53 in these cases and that there may be other cellular targets for HPV. The molecular mechanisms involved in the development and progression of lung cancer by HPV remain to be determined.

**Acknowledgements**

The authors thank Dr H zur Hausen for providing cloned HPV-16 and -18 DNA and Dr G Orth for providing cloned HPV-33 DNA. We also thank Professor H Kato of the Second Department of Surgery, Hokkaido University School of Medicine, Dr T Hirata of the National Sapporo Minami Hospital and Dr T Morikawa of the Sapporo Minami-ichijo Hospital for providing surgical specimens used in this study, and Professor T Yoshiki of the First Department of Pathology and Professor K Nagashima of the Second Department of Pathology, Hokkaido University School of Medicine, for providing autopsy specimens.

This work was supported in part by Grants-in-Aid for Cancer Research from the Ministry of Education, Science and Culture, and from the Ministry of Health and Welfare, Japan.

**References**

ANWAR K, NAKAKUKI K, NAIKI H AND INUZUKA M. (1993). ras gene mutations and HPV infection are common in human laryngeal carcinoma. *Int. J. Cancer*, **53**, 22–28.

BAND V, ZAJCHOWSKI D, KULESA V AND SAGER R. (1990). Human papilloma virus DNAs immortalize normal human mammary epithelial cells and reduce their growth factor requirements. *Proc. Natl Acad. Sci. USA*, **87**, 463–467.

BEJUI TF, LIAGRE N, CHIGNOL MC, CHARDONNET Y AND PATRICOT LM. (1990). Detection of human papillomavirus DNA in squamous bronchial metaplasia and squamous cell carcinomas of the lung by *in situ* hybridization using biotinylated probes in paraffin-embedded specimens. *Hum. Pathol.*, **21**, 111–116.

CHIBA I, TAKAHASHI T, NAU MM, D'AMICO D, CURIEL DT, MITSUDOMI T, BUCHHAGEN DL, CARBONE D, PIANTADOSI S, KOGA H, REISSMAN PT, SLAMON DJ, HOLMES EC AND MINNA JD. (1990). Mutations in the p53 gene are frequent in primary, resected non-small cell lung cancer. *Oncogene*, **5**, 1603–1610.

DE VILLIERS EM. (1989). Heterogeneity of the human papillomavirus group. *J. Virol.*, **63**, 4898–4903.

HARBOUR JW, LAI SL, WHANG PJ, GAZDAR AF, MINNA JD AND KAYE FJ. (1988). Abnormalities in structure and expression of the human retinoblastoma gene in SCLC. *Science*, **241**, 353–357.

HAWLEY NP, VOUSDEN KH, HUBBERT NL, LOWY DR AND SCHILLER JT. (1989). HPV16 E6 and E7 proteins cooperate to immortalize human foreskin keratinocytes. *EMBO J.*, **8**, 3905–3910.

HIGGINS GD, UZELIN DM, PHILLIPS GE, PIETERSE AS AND BURRELL CJ. (1991). Differing characteristics of human papillomavirus RNA-positive and RNA-negative anal carcinomas. *Cancer*, **68**, 561–567.

MANIATIS T, FRITSCH EF AND SAMBROOK J. (1989). *Molecular Cloning: A Laboratory Manual*, p. 9.16, Cold Spring Harbor Laboratory Press: Cold Spring Harbor, NY.

MUNGER K, WERNESS BA, DYSON N, PHELPS WC, HARLOW E AND HOWLEY PM. (1989). Complex formation of human papillomavirus E7 proteins with the retinoblastoma tumor suppressor gene product. *EMBO J.*, **8**, 4099-4105.

OSTROW RS, MANIAS DA, FONG WJ, ZACHOW KR AND FARAS AJ. (1987). A survey of human cancers for human papillomavirus DNA by filter hybridization. *Cancer*, **59**, 429-434.

PIETENPOL JA, STEIN RW, MORAN E, YACIUK P, SCHLEGEL R, LYONS RM, PITTELKOW MR, MUNGER K, HOWLEY PM AND MOSES HL. (1990). TGF-beta 1 inhibition of c-myc transcription and growth in keratinocytes is abrogated by viral transforming proteins with pRB binding domains. *Cell*, **61**, 777-785.

POPPER HH, WIRNSBERGER G, JÜTTNER-SMOLLE FM, PONGRATZ MG AND SOMMERSGUTTER M. (1992). The predictive value of human papilloma virus (HPV) typing in the prognosis of bronchial squamous cell papillomas. *Histopathology*, **21**, 323-330.

REISSMANN PT, KOGA H, TAKAHASHI R, FIGLIN RA, HOLMES EC, PIANTADOSI S, CORDON-CARDO C, SLAMON DJ AND GROUP TLCS. (1993). Inactivation of the retinoblastoma susceptibility gene in non-small-cell lung cancer. *Oncogene*, **8**, 1913-1919.

SCHEFFNER M, WERNESS BA, HUIBREGTSE JM, LEVINE AJ AND HOWLEY PM. (1990). The E6 oncoprotein encoded by human papillomavirus types 16 and 18 promotes the degradation of p53. *Cell*, **63**, 1129-1136.

SCHWARZ E, FREESE UK, GISSMANN LWM, ROGGENBUCK B, STREMLAU A AND ZUR HAUSEN H. (1985). Structure and transcription of human papillomavirus sequences in cervical carcinoma cells. *Nature*, **314**, 111-114.

SHAMANIN V, DELIUS H AND DE VILLIERS EM. (1994). Development of a broad spectrum PCR assay for papillomavirus and its application in screening lung cancer biopsies. *J. Gen. Virol.*, **75**, 1149-1156.

SHIMADA M, FUKUSHIMA M, MUKAI H, KATO I, NISHIKAWA A AND FUJINAGA K. (1990). Amplification and specific detection of transforming gene region of human papillomavirus 16, 18 and 33 in cervical carcinoma by means of the polymerase chain reaction. *Jpn J. Cancer Res.*, **81**, 1-5.

SHINDOH M, SAWADA Y, KOHGO T, AMEMIYA A AND FUJINAGA K. (1992). Detection of human papillomavirus DNA sequences in tongue squamous-cell carcinoma utilizing the polymerase chain reaction method. *Int. J. Cancer*, **50**, 167-171.

STOLER MH, MILLS SE, GERSELL DJ AND WALKER AN. (1991). Small-cell neuroendocrine carcinoma of the cervix. A human papillomavirus type 18-associated cancer. *Am. J. Surg. Pathol.*, **15**, 28-32.

STOLER MH, RHODES CR, WHITBECK A, WOLINSKY SM, CHOW LT AND BROKER TR. (1992). Human papillomavirus type 16 and 18 gene exprerssion in cervical neoplasias. *Hum. Pathol.*, **23**, 117-128.

STOREY A, PIM D, MURRAY A, OSBORN K, BANKS L AND CRAWFORD L. (1988). Comparison of the in vitro transforming activities of human papillomavirus types. *EMBO J.*, **7**, 1815-1820.

STREMLAU A, GISSMANN L, IKENBERG H, STARK M, BANNASCH P AND ZUR HAUSEN H. (1985). Human papillomavirus type 16 related DNA in an anaplastic carcinoma of the lung. *Cancer*, **55**, 1737-1740.

SYRJÄNEN K, SYRJÄNEN S, KELLOKOSKI J, KÄRJÄ J AND MÄNTYJÄRVI R. (1989). Human papillomavirus (H1PV) type 6 and 16 DNA sequences in bronchial squamous cell carcinomas demonstrated by *in situ* DNA hybridization. *Lung*, **167**, 33-42.

SZABO I, SEPP R, NAKAMOTO K, MAEDA M, SAKAMOTO H AND UDA H. (1994). Human papillomavirus not found in squamous and large cell lung carcinomas by polymerase chain reaction. *Cancer*, **73**, 2740-2744.

TAKAHASHI T, TAKAHASHI T, SUZUKI H, HIDA T, SEKIDO Y, ARIYOSHI Y AND UEDA R. (1991). The p53 gene is very frequently mutated in small-cell lung cancer with a distinct nucleotide substitution pattern. *Oncogene*, **6**, 1775-1778.

TAKEBE N, TSUNOKAWA Y, NOZAWA S, TERADA M AND SUGIMURA T. (1987). Conservation of E6 and E7 regions of human papillomavirus types 16 and 18 present in cervical cancers. *Biochem. Biophys. Res. Commun.*, **143**, 837-844.

TASE T, OKAGAKI T, CLARK BA, MANIAS DA, OSTROW RS, TWIGGS LB AND FARAS AJ. (1988). Human papillomavirus types and localization in adenocarcinoma and adenosquamous carcinoma of the uterine cervix: a study by *in situ* DNA hybridization. *Cancer Res.*, **48**, 993-998.

WERNESS BA, LEVINE AJ AND HOWLEY PM. (1990). Association of human papillomavirus types 16 and 18 E6 proteins with p53. *Science*, **248**, 76-79.

WILLEY JC, BROUSSOUD A, SLEEMI A, BENNETT WP, CERUTTI P AND HARRIS CC. (1991). Immortalization of normal human bronchial epithelial cells by human papillomavirus 16 or 18. *Cancer Res.*, **51**, 5370-5377.

WOODWORTH CD, BOWDEN PE, DONIGER J, PIRISI L, BARNES W, LANCASTER WD AND DIPAOLO JA. (1988). Characterization of normal human exocervical epithelial cells immortalized in vitro by papillomavirus types 16 and 18 DNA. *Cancer Res.*, **48**, 4620-4628.

YEE C, KRISHNAN-HEWLETT I, BAKER CC, SCHELEGEL R AND HOWLEY PM. (1985). Presence and expression of human papillomavirus sequences in human cervical carcinoma cell lines. *Am. J. Pathol.*, **119**, 361-366.

YOKOTA J, AKIYAMA T, FUNG YK, BENEDICT WF, NAMBA Y, HANAOKA M, WADA M, TERASAKI T, SHIMOSATO Y AND SUGIMURA T. (1988). Altered expression of the retinoblastoma (RB) gene in small-cell carcinoma of the lung. *Oncogene*, **3**, 471-475.

YOUSEM SA, OHORI NP AND SONMEZ AE. (1992). Occurrence of human papillomavirus DNA in primary lung neoplasms. *Cancer*, **69**, 693-697.

ZUR HAUZEN H. (1991). Viruses in human cancers. *Science*, **254**, 1167-1173.


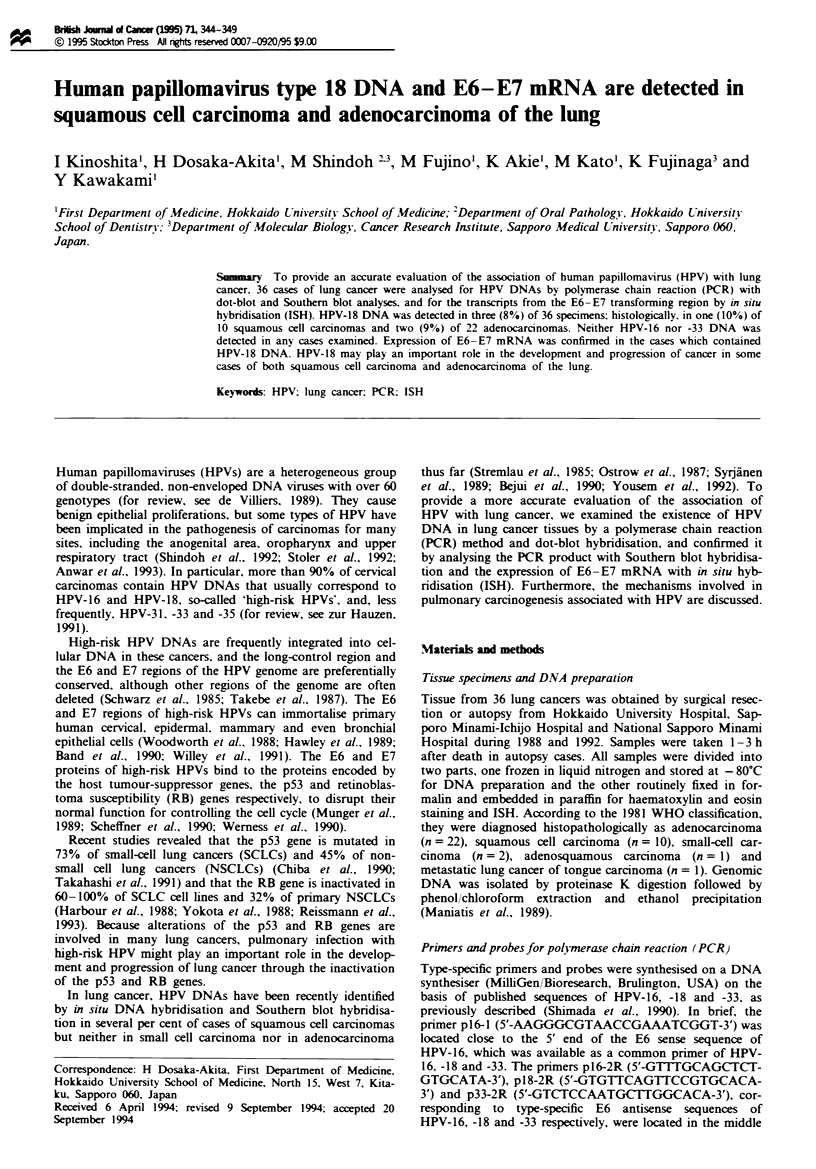

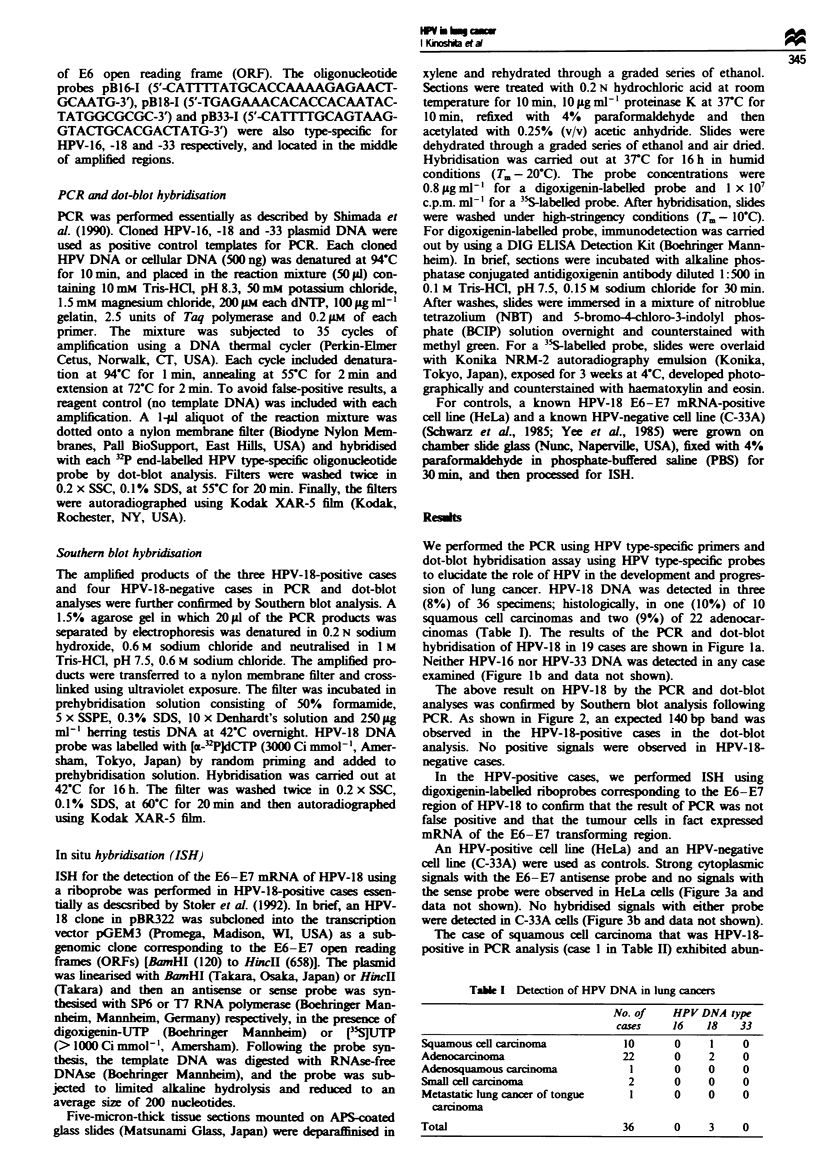

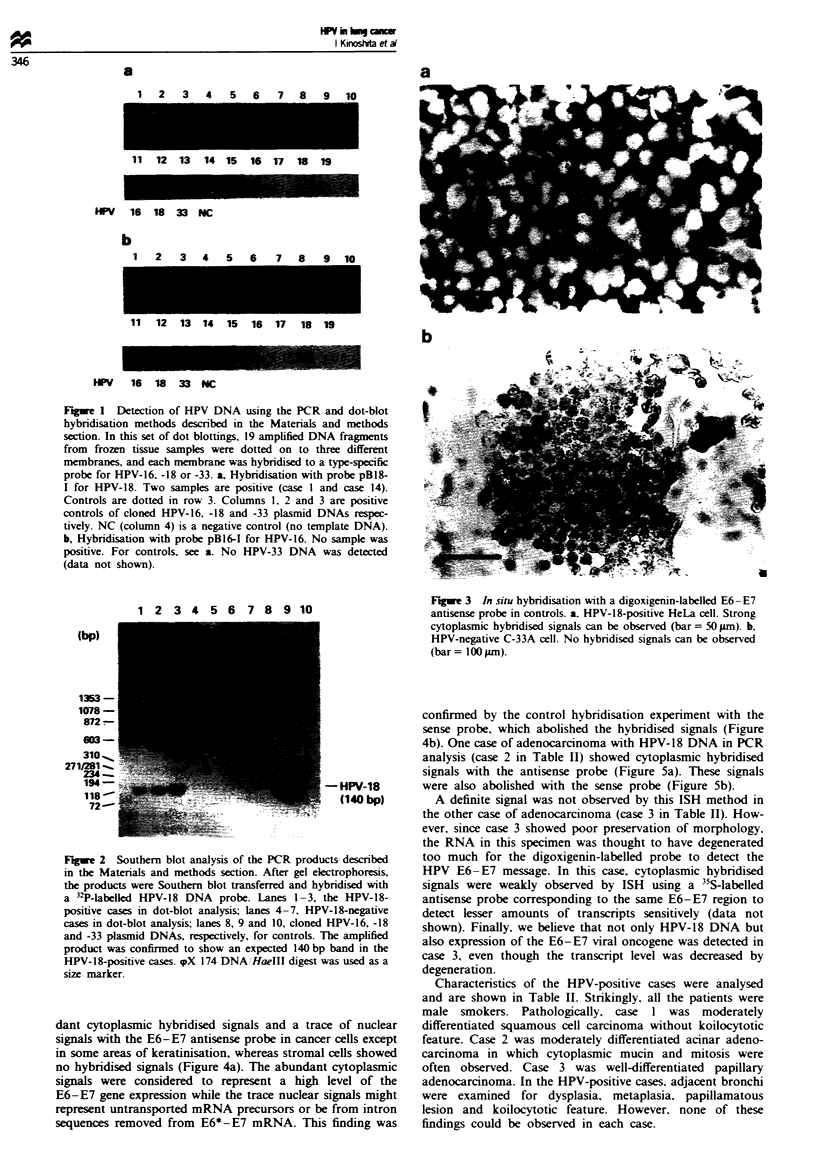

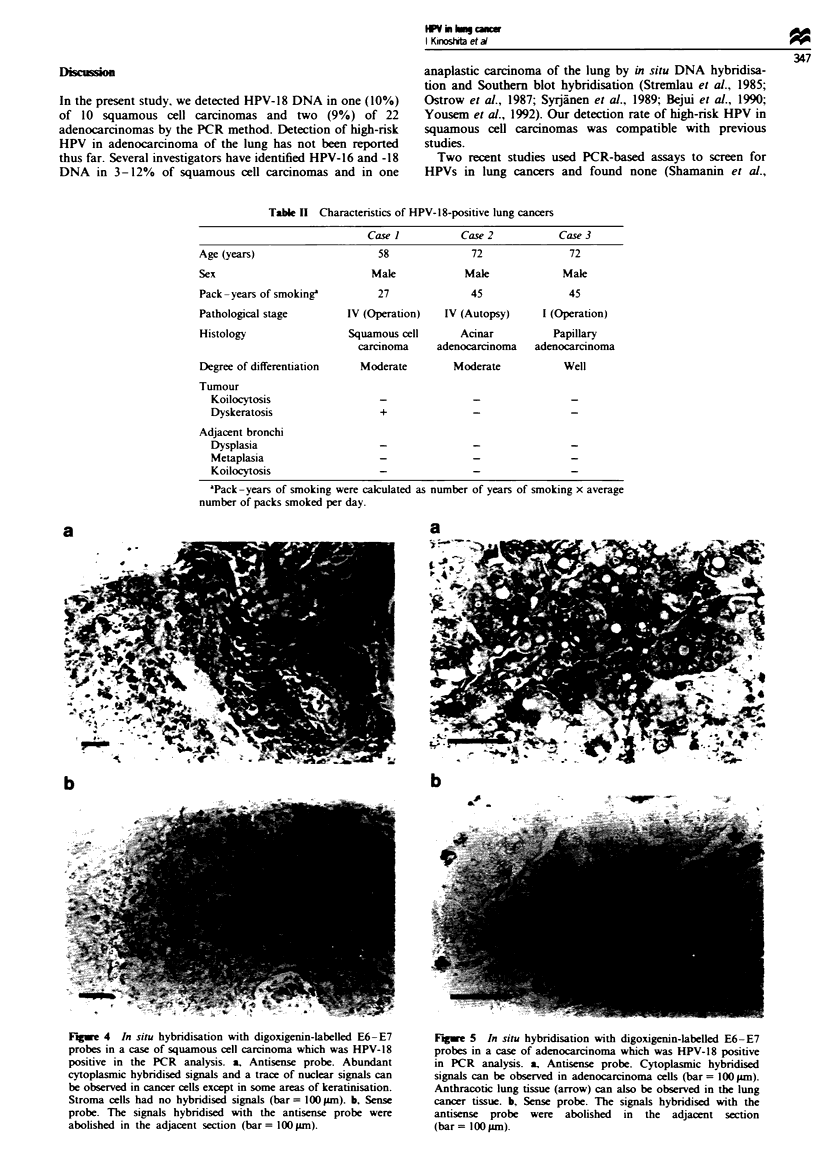

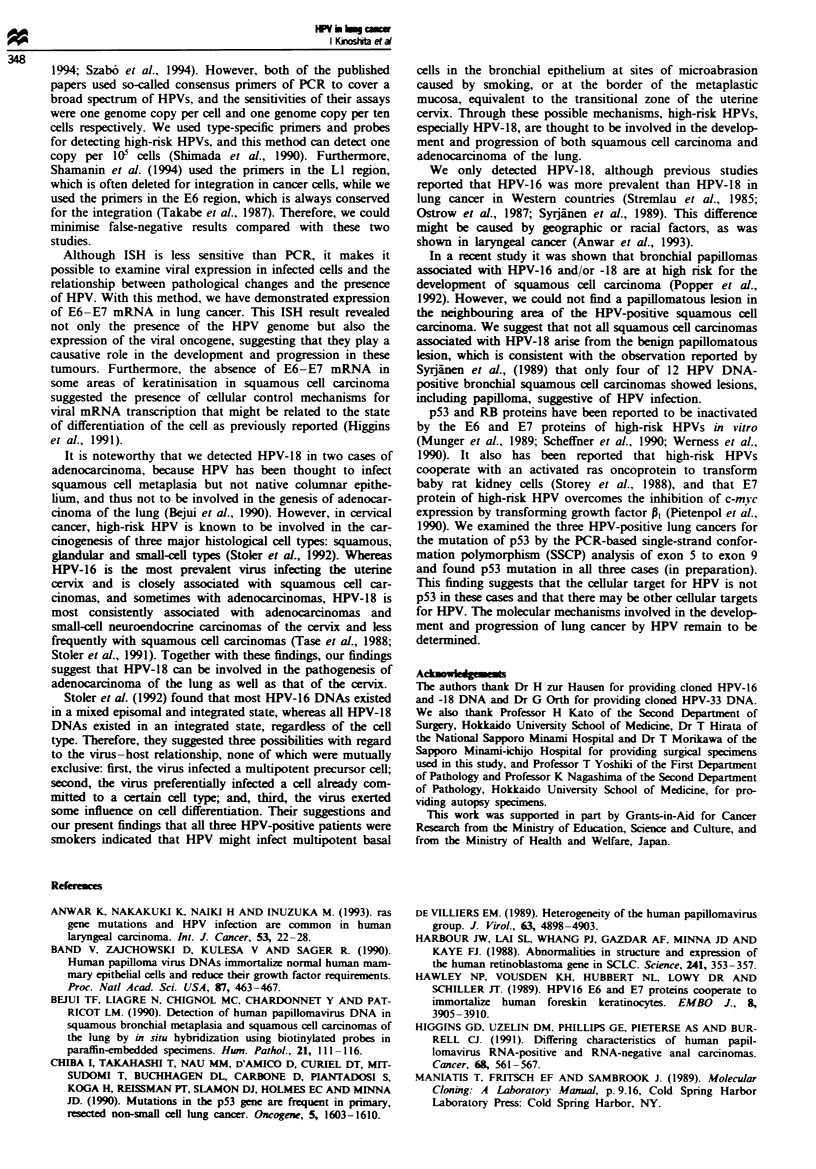

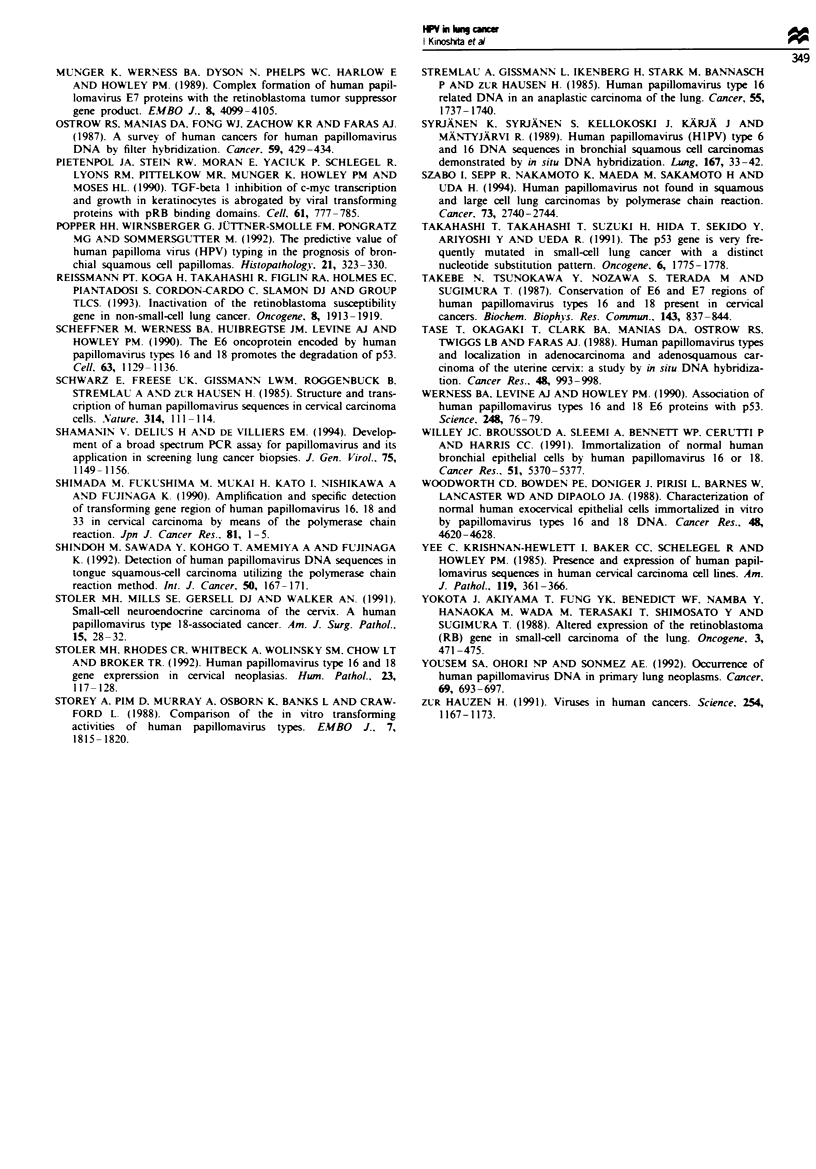


## References

[OCR_00618] Anwar K., Nakakuki K., Naiki H., Inuzuka M. (1993). ras gene mutations and HPV infection are common in human laryngeal carcinoma.. Int J Cancer.

[OCR_00623] Band V., Zajchowski D., Kulesa V., Sager R. (1990). Human papilloma virus DNAs immortalize normal human mammary epithelial cells and reduce their growth factor requirements.. Proc Natl Acad Sci U S A.

[OCR_00627] Béjui-Thivolet F., Liagre N., Chignol M. C., Chardonnet Y., Patricot L. M. (1990). Detection of human papillomavirus DNA in squamous bronchial metaplasia and squamous cell carcinomas of the lung by in situ hybridization using biotinylated probes in paraffin-embedded specimens.. Hum Pathol.

[OCR_00637] Chiba I., Takahashi T., Nau M. M., D'Amico D., Curiel D. T., Mitsudomi T., Buchhagen D. L., Carbone D., Piantadosi S., Koga H. (1990). Mutations in the p53 gene are frequent in primary, resected non-small cell lung cancer. Lung Cancer Study Group.. Oncogene.

[OCR_00645] Harbour J. W., Lai S. L., Whang-Peng J., Gazdar A. F., Minna J. D., Kaye F. J. (1988). Abnormalities in structure and expression of the human retinoblastoma gene in SCLC.. Science.

[OCR_00652] Hawley-Nelson P., Vousden K. H., Hubbert N. L., Lowy D. R., Schiller J. T. (1989). HPV16 E6 and E7 proteins cooperate to immortalize human foreskin keratinocytes.. EMBO J.

[OCR_00655] Higgins G. D., Uzelin D. M., Phillips G. E., Pieterse A. S., Burrell C. J. (1991). Differing characteristics of human papillomavirus RNA-positive and RNA-negative anal carcinomas.. Cancer.

[OCR_00673] Münger K., Werness B. A., Dyson N., Phelps W. C., Harlow E., Howley P. M. (1989). Complex formation of human papillomavirus E7 proteins with the retinoblastoma tumor suppressor gene product.. EMBO J.

[OCR_00679] Ostrow R. S., Manias D. A., Fong W. J., Zachow K. R., Faras A. J. (1987). A survey of human cancers for human papillomavirus DNA by filter hybridization.. Cancer.

[OCR_00685] Pietenpol J. A., Stein R. W., Moran E., Yaciuk P., Schlegel R., Lyons R. M., Pittelkow M. R., Münger K., Howley P. M., Moses H. L. (1990). TGF-beta 1 inhibition of c-myc transcription and growth in keratinocytes is abrogated by viral transforming proteins with pRB binding domains.. Cell.

[OCR_00691] Popper H. H., Wirnsberger G., Jüttner-Smolle F. M., Pongratz M. G., Sommersgutter M. (1992). The predictive value of human papilloma virus (HPV) typing in the prognosis of bronchial squamous cell papillomas.. Histopathology.

[OCR_00695] Reissmann P. T., Koga H., Takahashi R., Figlin R. A., Holmes E. C., Piantadosi S., Cordon-Cardo C., Slamon D. J. (1993). Inactivation of the retinoblastoma susceptibility gene in non-small-cell lung cancer. The Lung Cancer Study Group.. Oncogene.

[OCR_00701] Scheffner M., Werness B. A., Huibregtse J. M., Levine A. J., Howley P. M. (1990). The E6 oncoprotein encoded by human papillomavirus types 16 and 18 promotes the degradation of p53.. Cell.

[OCR_00709] Schwarz E., Freese U. K., Gissmann L., Mayer W., Roggenbuck B., Stremlau A., zur Hausen H. (1985). Structure and transcription of human papillomavirus sequences in cervical carcinoma cells.. Nature.

[OCR_00715] Shamanin V., Delius H., de Villiers E. M. (1994). Development of a broad spectrum PCR assay for papillomaviruses and its application in screening lung cancer biopsies.. J Gen Virol.

[OCR_00426] Shimizu S., Sabsay B., Veis A., Ostrow J. D., Rege R. V., Dawes L. G. (1989). Isolation of an acidic protein from cholesterol gallstones, which inhibits the precipitation of calcium carbonate in vitro.. J Clin Invest.

[OCR_00722] Shindoh M., Sawada Y., Kohgo T., Amemiya A., Fujinaga K. (1992). Detection of human papillomavirus DNA sequences in tongue squamous-cell carcinoma utilizing the polymerase chain reaction method.. Int J Cancer.

[OCR_00734] Stoler M. H., Mills S. E., Gersell D. J., Walker A. N. (1991). Small-cell neuroendocrine carcinoma of the cervix. A human papillomavirus type 18-associated cancer.. Am J Surg Pathol.

[OCR_00740] Stoler M. H., Rhodes C. R., Whitbeck A., Wolinsky S. M., Chow L. T., Broker T. R. (1992). Human papillomavirus type 16 and 18 gene expression in cervical neoplasias.. Hum Pathol.

[OCR_00746] Storey A., Pim D., Murray A., Osborn K., Banks L., Crawford L. (1988). Comparison of the in vitro transforming activities of human papillomavirus types.. EMBO J.

[OCR_00753] Stremlau A., Gissmann L., Ikenberg H., Stark M., Bannasch P., zur Hausen H. (1985). Human papillomavirus type 16 related DNA in an anaplastic carcinoma of the lung.. Cancer.

[OCR_00759] Syrjänen K., Syrjänen S., Kellokoski J., Kärjä J., Mäntyjärvi R. (1989). Human papillomavirus (HPV) type 6 and 16 DNA sequences in bronchial squamous cell carcinomas demonstrated by in situ DNA hybridization.. Lung.

[OCR_00763] Szabó I., Sepp R., Nakamoto K., Maeda M., Sakamoto H., Uda H. (1994). Human papillomavirus not found in squamous and large cell lung carcinomas by polymerase chain reaction.. Cancer.

[OCR_00770] Takahashi T., Takahashi T., Suzuki H., Hida T., Sekido Y., Ariyoshi Y., Ueda R. (1991). The p53 gene is very frequently mutated in small-cell lung cancer with a distinct nucleotide substitution pattern.. Oncogene.

[OCR_00775] Takebe N., Tsunokawa Y., Nozawa S., Terada M., Sugimura T. (1987). Conservation of E6 and E7 regions of human papillomavirus types 16 and 18 present in cervical cancers.. Biochem Biophys Res Commun.

[OCR_00781] Tase T., Okagaki T., Clark B. A., Manias D. A., Ostrow R. S., Twiggs L. B., Faras A. J. (1988). Human papillomavirus types and localization in adenocarcinoma and adenosquamous carcinoma of the uterine cervix: a study by in situ DNA hybridization.. Cancer Res.

[OCR_00788] Werness B. A., Levine A. J., Howley P. M. (1990). Association of human papillomavirus types 16 and 18 E6 proteins with p53.. Science.

[OCR_00794] Willey J. C., Broussoud A., Sleemi A., Bennett W. P., Cerutti P., Harris C. C. (1991). Immortalization of normal human bronchial epithelial cells by human papillomaviruses 16 or 18.. Cancer Res.

[OCR_00800] Woodworth C. D., Bowden P. E., Doniger J., Pirisi L., Barnes W., Lancaster W. D., DiPaolo J. A. (1988). Characterization of normal human exocervical epithelial cells immortalized in vitro by papillomavirus types 16 and 18 DNA.. Cancer Res.

[OCR_00806] Yee C., Krishnan-Hewlett I., Baker C. C., Schlegel R., Howley P. M. (1985). Presence and expression of human papillomavirus sequences in human cervical carcinoma cell lines.. Am J Pathol.

[OCR_00810] Yokota J., Akiyama T., Fung Y. K., Benedict W. F., Namba Y., Hanaoka M., Wada M., Terasaki T., Shimosato Y., Sugimura T. (1988). Altered expression of the retinoblastoma (RB) gene in small-cell carcinoma of the lung.. Oncogene.

[OCR_00817] Yousem S. A., Ohori N. P., Sonmez-Alpan E. (1992). Occurrence of human papillomavirus DNA in primary lung neoplasms.. Cancer.

[OCR_00641] de Villiers E. M. (1989). Heterogeneity of the human papillomavirus group.. J Virol.

[OCR_00822] zur Hausen H. (1991). Viruses in human cancers.. Science.

